# Microbial Pathogenicity in Space

**DOI:** 10.3390/pathogens10040450

**Published:** 2021-04-09

**Authors:** Marta Filipa Simões, André Antunes

**Affiliations:** 1 State Key Laboratory of Lunar and Planetary Sciences (SKLPlanets), Macau University of Science and Technology (MUST), Avenida Wai Long, Taipa, Macau, China; aglantunes@must.edu.mo; 2 China National Space Administration (CNSA), Macau Center for Space Exploration and Science, Macau, China

**Keywords:** space exploration, microgravity, microorganisms, pathogens

## Abstract

After a less dynamic period, space exploration is now booming. There has been a sharp increase in the number of current missions and also of those being planned for the near future. Microorganisms will be an inevitable component of these missions, mostly because they hitchhike, either attached to space technology, like spaceships or spacesuits, to organic matter and even to us (human microbiome), or to other life forms we carry on our missions. Basically, we never travel alone. Therefore, we need to have a clear understanding of how dangerous our “travel buddies” can be; given that, during space missions, our access to medical assistance and medical drugs will be very limited. Do we explore space together with pathogenic microorganisms? Do our hitchhikers adapt to the space conditions, as well as we do? Do they become pathogenic during that adaptation process? The current review intends to better clarify these questions in order to facilitate future activities in space. More technological advances are needed to guarantee the success of all missions and assure the reduction of any possible health and environmental risks for the astronauts and for the locations being explored.

## 1. Introduction

Despite their small dimensions, microbes have an enormous impact both on the environments they inhabit and on a global scale. On Earth, they clearly impact climate regulation, being responsible for half of total CO_2_ fixation and for the establishment and maintenance of an oxygen-rich atmosphere [1]. Microbes can also have very impactful interactions with host organisms, but at a different scale. At both levels, the interplay between microbes and their environments creates a complex network of interactions and effects.

Our understanding of microbial interactions and impact is being pushed into new frontiers, extending beyond the confines of our own planet. Space exploration is witnessing increasingly longer crewed missions, with astronauts expected to spend extended time in spaceflights, especially with planned missions to the Moon and Mars, which raises several challenges and potential hazards [2,3,4,5,6].

Urgent technological advances are necessary to reduce health risks to astronauts during their missions. All necessary security and safety measurements need to be considered beforehand because any occurring mission improvements will have to happen on board, with all the limitations of space vehicles [2]. Such advances should focus on detection and control of microbes and microbial diversity on astronauts and spacecraft [7], as well as on monitoring of spacecraft water systems and ensuring the identification and counteract measures of any waterborne microbial contamination [3]. Linked to both of these aspects, some authors suggest microbiome manipulation as a way to guarantee and improve astronauts’ health [7].

A significant research emphasis has been placed on assessing physiological and phenotypic changes of microbes (and astronauts) when exposed to the space environment or space-like conditions, as portrayed by Criscuolo et al. [5]. As stated by these authors, deep space exploration might become longer, and the exposure to the space environment will lead to adaptations relevant to fields such as evolutionary biology and ecology [5]. Nevertheless, information on multiple combined stressors or on complex microbial communities is still lacking.

### 1.1. How Do Microorganisms Get to Space?

The traffic and transit of microbes in and into space has received some attention and might be associated with multiple sources. The majority of the research done so far on this topic is based on the International Space Station (ISS) microbiology experiments [3]. Here, in low Earth orbit (LEO; at around 400 km from Earth’s surface) [8], life is somewhat protected by the Earth’s magnetosphere [9]. Further space exploration, beyond LEO, will be even less accommodating, but the ISS offers unique insights into life in space. In an attempt to understand the origin of microbial contaminations, the microbiomes of the ISS and the commercial resupply vehicle (CRV) have been compared. Only a limited amount of ISS microbes was found to have the CRV as their source; in fact, the CRV’s microbiome was quite different and had a much lower number of microorganisms. This proves the successful performance of cleaning protocols for CRV surfaces and points to the crew as the likely source of the ISS’s diverse microbiome [10]. Most microorganisms found in the ISS are normally perceived as not relevant nor causing disease; however, with long exposure to microgravity, the astronauts’ immune systems tend to be less responsive and might struggle to fight these microorganisms, which would then become pathogenic [3]. As an additional factor, the risk of becoming infected with a pathogenic microorganism is increased in confined spacecraft environments and with the close proximity of astronauts [7,11,12]. Further risks and restrictions include access to treatment options, drugs, and resources, as well as the ability to perform proper hygiene [7].

Microbial presence and movement across different layers of our atmosphere is another relevant aspect. Aerobiology has shown that microorganisms can be transferred in the air through aerosols or attached to other particles. After this discovery, studies at higher altitudes were among some of the pioneer studies in the field of exobiology and astrobiology [13]. It is now known that many microorganisms survive in our atmosphere, with reports of maintenance of viability of pathogenic strains after exposure to the stratosphere [8,13]. Microbial isolations have been successfully performed from air and dust samples from the troposphere and stratosphere [8], and many others have been made from space station such as those described in [14,15,16,17,18].

As an additional source, and according to the panspermia hypothesis, microorganisms can be transferred across space between different parts of the solar system [8,19]. This theory achieved considerable traction after the discovery of Martian meteorites, probably ejected from their original location after large impacts [20]. Lithopanspermia further defends that microbial transference can occur through the protection and shielding against ultraviolet (UV) radiation by material such as rocks [8,20]. More recently, Kawaguchi et al. (2013), proposed massapanspermia, based on protective properties of non-superficial cells when clustered in aggregates [21]. This protective effect, which would facilitate survival in space, could be due to layers of UV-inactivated cells shielding underlying cells, as observed with experiments with *Bacillus subtilis* spores [20].

### 1.2. How Can We Study the Potential Pathogenicity of Microorganisms in Space Exploration?

**Figure 1:**
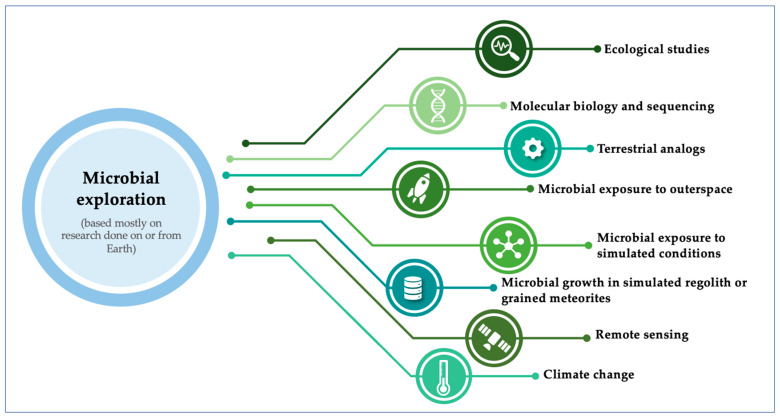
Potential methodological tools to explore possible pathogens in space exploration.

There are many approaches to understand the potential of a microorganism in becoming pathogenic. To reach this goal in a space exploration context, we need to study microbial interactions related to astrobiological studies of microorganisms, through several methodological approaches (Figure 1).

#### 1.2.1. Ecological Studies

One of these approaches is the use of ecological studies through e.g., landscape genetics (a combination of the study of population genetics with landscape ecology), to better understand pathogen dynamics and disease ecology [22]. The combination of ecological concepts with astrobiological analysis, is an example of a multidisciplinary approach that can facilitate microbial research in astrobiology. Examples of these are the studies published by Meslier and DiRuggiero, which explain the relation between the research of the limits of life and potential habitability with the study of microbial communities from lithic environments [23] and the studies by Méndez et al., which propose the use of habitability models for common application of ecological and astrobiological research [24]. Over the last few years there has been an increase of these studies [25,26,27], from in silico analysis with modelling and simulations, to genomic and phylogenetic approaches and the use of genetic markers to understand pathogen evolution and their interactions with their respective hosts [22].

#### 1.2.2. Molecular Biology and Sequencing

Another approach to fully understand the existing microbes and their dynamics, inside spacecrafts, on astronauts, and on anything going in or out of the spacecrafts, is the use of molecular biology and sequencing experiments. For this, swabbing of the surfaces and the astronauts’ bodies, over a defined period of time, followed with direct DNA extraction, or microbial isolation and DNA extraction, and sequencing for identification of the microbiomes is the most common method, which is what was done for example by Mahnert et al. [7] and Sielaff et al. [16]. Although frequently neglected, morphological and physiological characterization of the isolates also helps to understand any phenotypic changes after isolation and provides very important insights and material for further investigations.

#### 1.2.3. Terrestrial Analogues

The study of microbial life and adaptation in terrestrial analogues is another useful approach. The exploration of these analogues (locations with conditions similar to those present in other parts of our solar system) is one of the main pillars of astrobiology and, despite some well-recognized limitations, provides vital insights into planning future missions [28,29,30]. We are still several years away from having direct access to samples from Mars, Europa, Enceladus or other locations of astrobiological relevance; until then, we are limited to using this type of approach to test and refine equipment, methodologies, and techniques, as well as better understand the limits of life and get a more accurate view of resilience, long-term viability of microbes and biomolecules, biodiversity, and adaptations to extreme settings [28,30,31]. Increased understanding of these aspects is also expected to contribute to a better estimation of the real risk of potential pathogenic microbes and eventual control measures under space conditions.

#### 1.2.4. Microbial Exposure to Outer Space

A further method, which has been extensively explored, is the use of microbial exposure to outer space conditions or simulated outer space conditions. This has been performed many times to test for survivability and as a way of assessing the panspermia hypothesis. For several decades, microbial samples of all kind (bacteria, bacterial spores, fungi, fungal spores, cyanobacteria, algae, phages and DNA) have been exposed in balloons, rockets, and spacecrafts in order to undertake the first experiments of astrobiology [19]. For example, during the Tanpopo mission, Kawaguchi et al. exposed dried deinococcal cell pellets, for a period of up to three years, at the Exposure Facility of the Japanese Experimental Module (JEM) of the ISS [8]. The cells were analysed at several time points during the experiment to check for survivability and for any other changes [8].

#### 1.2.5. Microbial Exposure to Simulated Conditions

These types of project, which are essential to our understanding of life development under outer space conditions, are still very difficult to perform. There are limited locations to do this, limited opportunities due to the relatively low number of missions (even though these are increasing), they are associated with prohibitive costs, and there are many volume and weight limitations [17].

Alternatively, many studies are performed in simulated microgravity conditions on Earth [17]. There are several different systems used for this purpose: the random positioning machine (RPM) or 3D clinostat, the clinostat, and the rotating wall vessel (RWV) [32]. The RWV bioreactor can have a high aspect ratio vessel (HARV), or a slow-turning lateral vessel (STLV), depending on the type of aeration which can be through a gas permeable membrane for HARV, or a central core gas exchange membrane for the STLV [33,34]. Within these, the HARV and the clinostat are appropriate for liquid assays, and the clinostat can also be used for solid media assays [32,33,34].

However, any simulation system on Earth is not able to fully mimic the multifactorial conditions of a space flight. Regardless, this is still a more affordable and less complex way (“in terms of experiment size, weight, electric power requirements and so on” [35]) to analyse specific phenotypic traits and physiological mechanisms altered over spaceflight [35]. However, when performing research with simulated microgravity systems, it is important to consider that different systems, like RWV and RPM [36], are good alternatives to real space gravity, but might have different impact and result in different cell response [37].

A few research groups have also made important progress in the use of different types of simulation chamber, with the capability to replicate different aspects of exposure to space or the surface of different moons and planets of the solar system [38]. For example, Olsson-Francis et al. used a set of high-pressure flow-through reactors to simulate sub-surface Martian and icy moon environments [39], and Martin and Cockell described a simulation chamber (PELS: Planetary Environmental Liquid Simulator) that simulates the conditions present in Martian water environments [40].

Exposure-based testing of microbes representing different taxa is essential to obtain a rough overview of general trends in microbial adaptations, resilience, and overall changes in the gene expression, physiology, and pathogenicity of different microbes when subjected to single and multiple stress sources linked with space exploration. The significant knowledge gaps in this field constitute an obvious threat both from a planetary protection and from a human health perspective, conditioning future missions.

#### 1.2.6. Microbial Growth in Simulated Regoliths or Grained Meteorites

Testing microbial survival and development when exposed to and grown in different substrates and media, such as simulated Martian dust or regoliths, similar in chemical composition, particle size, density, porosity, and magnetic properties to surface soils on Mars, derived from certain earth locations, is one way of testing the potential for microbes to survive on Mars [41]. Examples of simulated Martian dust or regoliths include: grained volcanic palagonite from Hawaii [42]; clay from Adendorf, Germany; red sandstone from Heidelberg, Germany; or one of several artificial Martian soil simulants, e.g., a mineral-based Mars Global Simulant (MGS-1) [43], simulants of four Martian environments (early basaltic terrain, sulphur-rich regolith, haematite-rich regolith and contemporary Mars regolith) [44], and different types of Mars soil analogues [45]. Microbial studies have also been undertaken with grained meteorites such as the Millbillillie meteorite, probably from the asteroid Vesta; the Martian meteorite Zagami, and the Kaba meteorite [19,45,46].

#### 1.2.7. Remote Sensing

Studying microorganisms, using space-based technologies, is another approach that can be undertaken via remote sensing with satellites. For example, satellites can be used to detect surface microbial by-products on Earth, such as: chlorophyll a (indicative of oxygenic photosynthesis), heterotrophic prokaryotic production, or volatile compounds like reduced sulphur compounds (e.g., dimethyl sulphide) emitted from the oceans on Earth. In short, remote sensing works by sending a beam of radiation to a pre-determined target, or uses the sun as the initial stimulatory radiation source, and a sensor detects the radiation sent back from the target allowing researchers to infer and estimate microbial mechanisms and activities [1].

#### 1.2.8. Climate Change

Studying microbial evolution associated with climate change is a way of understanding the adaptation mechanisms of microbes, when facing changes in abiotic factors, such as: elevated temperature, increased CO_2_, increased salinity and altered water availability [47]. It is worth noting that there are some on-going discussions focused on how climate change is likely leading to increased health issues, namely by changes in microbial biodiversity and distribution, in microbial composition and function, in microbial physiological responses with phenotypic shifts and evolutionary adaptations, as well as cases of increased pathogenicity and potentially depressed immunity [6,48,49,50,51,52]. It is now known that climate change aggravates the global spread of pathogens (vector borne, foodborne, airborne, waterborne and other environmental pathogens) and their associated diseases, stresses marine life causing disease and disrupting regular ecosystem functions, and increases antimicrobial resistance of microorganisms, threatening life on Earth, human health, and food security [53]. Parallels with space-flight stress and its effects on pathogenicity and health in space might prove quite helpful, and many of the technological systems used to analyse changes in climate also have applicability in space exploration, like satellites for meteorological analysis or for water distribution [54].

### 1.3. How do Microorganisms React to Space Conditions?

Space conditions include a combination of environmental stressors, which can have a detrimental effect on microbial populations. However, some microorganisms develop different phenotypes and metabolic activities when exposed to stressful conditions and environments. Space parameters greatly influence survival and genetic stability of microorganisms, impacting on their distribution and evolution [20]. A few of them adapt to the unique stresses they encounter, by changing some of their phenotypical characteristics to try to gain some selective advantage. An example of this is seen for fungi and their production of secondary metabolites [18].

In general, microbial organisms interact (each in specific ways) with extreme environmental stressors: gravity, pressure, temperature and oxygen, and develop ways to survive them [4]. Microbial studies in spaceflights and space missions, report that common microorganisms lose their viability when fully exposed to solar UV radiation and space vacuum, however some survive space exposure [55], and many show potentially relevant changes from a pathogenicity perspective. These include variations of growth kinetics (decreased lag phase and increased exponential phase), metabolic changes in the production of secondary metabolites, reduced antimicrobial sensitivity, and amplified contamination rates [12]. Most human pathogenic microorganisms are mesophilic and tend to struggle when exposed to temperatures higher than regular human body temperature [56] or more generally to very extreme conditions. In space, temperature can vary significantly. As an example, associated with the ISS, we have settings of controlled temperature (between 21 and 23 °C) inside the station, and a large range of varying temperature (from −100 to over 100 °C), on space-exposed surfaces [57].

Additionally, the cytoskeleton systems, constituted of filamentous proteins that go from the nucleus to the cell membranes, organize and direct the cellular growth and division [58]. It has been suggested that in prokaryotic cells, such systems would be responsible for mechanosensing and mechanotransduction, and for complex signal transduction networks that modulate their genetics in response to microgravity, in a similar way to what has been observed in eukaryotic cells [58]. These systems and their potential downstream effects would thus constitute an additional, underexplored factor, which should be taken into consideration.

Microbial populations are dynamic rather than static, which also introduces an extra layer of complexity to the system, further distancing reality from the results of controlled, simple experiments. Microbes in space are diverse and shift and change over time [16]. Furthermore, pathogens are fast evolving to keep their advantages over their hosts, tend to have shorter generation times, and often experience stronger selection [59]. The stressors found in outer space are parameters that induce several changes (Table 1) that can grant microorganisms an adaptation to new surroundings.

#### 1.3.1. Bacteria

Sielaff et al. reported that more than 100 bacterial strains were isolated from surface wipes, collected at eight different locations inside the ISS, over a period of 14 months, during 3 flight missions [16]. Those isolates were identified by Sanger sequencing of the 16S rRNA gene and *Staphylococcus* was the genus with the highest number of isolates, followed by *Pantoea* and *Bacillus*. The most commonly isolated species were *Staphylococcus aureus*, *Pantoea conspicua* and *Pantoea gaviniae* [16]. *S. aureus* is a Gram-positive pathogen responsible for several different infections in humans [61]. *P. gaviniae* [62] and *P. conspicua* [63] were only isolated and described a decade ago. They are both facultative anaerobic Gram-negative bacteria and, though seemingly ubiquitous, these particular species have not been associated with human infections [64].

Less abundant, but also detected in the aforementioned study, were several *Klebsiella* spp. isolates retrieved from various locations on ISS environmental surfaces: *K. aerogenes*, *K. pneumoniae*, and *K. quasipneumoniae* [16]. *K. pneumoniae* is often related with human infections, therefore, further analysis of their genomes [65] might shed some light on any metabolic and genetic adaptations derived from ISS environment exposure.

Another relevant microbe, *Serratia marcescens*, is a human opportunistic bacterium that has been previously isolated from the Mir spacecraft and from condensed water from the ISS [17]. Lately, there has been an increase in notifications of nosocomial *S. marcescens* infections [66,67]. This makes this species a potential hazard for space missions, as it might take advantage of any immunocompromised astronauts. Any possible space-induced changes should be thoroughly analysed [17].

Interestingly, DNA from bacteria of the genus *Mycobacteria* and the genus *Delftia* (family *Comamonadaceae*, order *Burkholderiales*) were found in samples of cosmic dust collected from the surface of the illuminator of the ISS, possibly transferred from the stratosphere into the ionosphere where the ISS is located [68]. Mycobacteria are a major concern, as some species can cause human infections, which are often hard to treat [69]. Some of those pathogenic bacteria can be found in water-related systems, in the form of biofilms. Grown biofilms have been found on several space stations: Salyut, Mir, Skylab, and the ISS [70]. For example, *Mycobacterium hassiacum*, is a fast-growing mycobacterium, often found in biofilms of showerheads. It is the most thermophilic non-tuberculous mycobacteria known so far. It is able to grow at high temperatures, up to 65 °C, and it is still viable at temperatures close to pasteurization values [56]. However, this mycobacterium has been mentioned in some reports as being the cause of some infections in humans. Even though not clinically relevant at the moment, this proves its tolerance to thrive in a very wide range of temperatures [56,71]. In order to shed some light on mycobacteria in space, Abshire et al. [72] exposed *Mycobacterium marinum* to low-shear modelled microgravity (an environment that simulates a nutrient-deprived environment and mimics the conditions inside macrophages during infection). They found the mycobacterial cells to have a decreased translation rate, downregulated genes involved in metabolic pathways, increased lipid degradation, and upregulated genes responsible for chaperone and mycobactin expression. These alterations, if happening on a pathogenic or potential pathogenic mycobacterial species, can lead to increased virulence and become a health risk to astronauts [72].

*Bacillus subtilis*, is a very well-studied bacterial species, used as a model for many research studies for being spore-forming and having unique characteristics (e.g., producer of many enzymes and secondary metabolites, involved in fermentation of several food products, with surface motility, forms biofilms and is even able to attach to plant root or fungal hyphae, and naturally competent) [73]. This species was tested for survival under high UV irradiation and simulated Martian conditions in a Mars simulation chamber (MSC). Several combinations of Martian conditions (different values of: gas composition, temperatures, ultraviolet–visible (UV-VIS) light, pressure) were tested under different timings, and it was found that its endospores survived irradiation under a certain degree of protection from full exposure, but survived better with reduced UV [41]. Besides this species, other representatives of the genus have also been isolated from swabs of different surfaces of the ISS: *B. pumilus*, *B. licheniformis*, *B. megaterium*, and *B. amyloliquefaciens*, with *B. pumilus* appearing to be the most resistant to radiation and dehydration [14].

*Escherichia coli* and *Serratia liquefaciens*, two faecal environment contaminants, able to replicate under low atmospheric pressures of 2.5 kPa, were found unable to grow under simulated Mars conditions, in a Mars analogue soil, grained volcanic palagonite from Hawaii. However, even though *E. coli* cells were not able to grow, replicate or reproduce, they managed to keep some viability after a ≈7 days exposure [42]. In a different study, *E. coli*, grown on the ISS (exposed to microgravity) with increasing concentrations of gentamicin, was analysed regarding its transcriptomic response [74]. When compared to controls on Earth, it was found that the ISS bacteria had an increased adaptation to the higher antibiotic concentrations, with upregulation of 50 genes related to stress responses, as well as activation of mechanisms related to oxidative stress and starvation [74]. The authors of this study suggested that the differences observed in space were due to the exposure to microgravity, stressing nutrient-deprived and acid-shock conditions [74].

#### 1.3.2. Fungi

Fungi are incredibly adaptable to environmental stress conditions, resulting in a wide range of changes in gene regulation, enzymatic activity and secondary metabolite production. All of these could affect pathogenicity and their health impact and are thus directly relevant to our discussion on space environment and a great part of the focus of astromycology.

Exposure to increased temperatures and UV radiation can cause fungi to mutate and change their toxigenic and mycotoxigenic profiles [47]. We currently know that, when facing such changes, certain fungal species adapt by shifting their genetic regulation (activating and downregulating certain genes), altering interactions with hosts and host-resistance [47]. These changes are having an increasingly significant impact on agriculture and health on Earth, as they can affect food and feed products, and increase mycotoxins’ contamination issues [47].

Desiccation is another environmental stressor which induces significant changes in fungi. As an example, when grown in NaCl-saturated concentrations and becoming extremely water-deprived (due to reduced water-activity—a_w_), *Aspergillus sydowii* suffers morphological alterations (unpolarised and highly septated hyphae with increased multinucleation), increased antioxidant enzymes production, upregulated genes, and oxidative stress [75].

Through transcriptomic analyses, it was possible to correlate the exposure of *Aspergillus flavus* to abiotic stressors, with ensuing increased expression of biosynthetic genes, increased production of secondary metabolites, increased growth and increased hazardous potential [76]. *Aspergillus flavus*, the second most common cause for human aspergillosis [77], is a saprophytic soil fungus and a contaminant of preharvest and postharvest seed crops [78] that, when exposed to increased temperature and lowered a_w_, presented upregulation of the genes responsible for the production of aflatoxins, leading to increased aflatoxin B_1_ production and growth stimulation [76].

Many fungal species have also been found as surviving in space environments. This was the case of more than 30 filamentous fungal species within the ISS, identified by Vesper et al. [79]. Among those, several potential opportunistic pathogens (*A. flavus* and *A. niger*) and potential moderate toxin producers (*Penicillium chrysogenum* and *Penicillium brevicompactum*) were encountered, which can become a health hazard to the astronauts [79]. Worryingly, *Aspergillus fumigatus*, a common cause of fungal infections, was also detected in this and in later surveys [16,79]. When the ISS *A. fumigatus* isolates from Vesper et al. [79] were analysed, an increased radial growth rate was observed, and even though DNA damage was expected (due to the prolonged time inside the ISS), no chromosomal aberrations or mutations stood out from the expected ones that were noted. Despite the lack of a control isolate for comparison, when compared to clinical isolates of the same species, the ISS strains were more resistant to UV irradiation, had different profiles of production of secondary metabolites, and were more virulent [80]. Blachowicz et al., also noted changes and molecular adaptations in *A. fumigatus* caused by the uncommon ISS environment: alterations of the proteome and increased number of proteins involved in stress responses, and carbohydrate and secondary metabolism [15].

*Aspergillus fumigatus* is a well-studied example of a successful pathogen, as it is the most significant airborne opportunistic pathogenic mould [80], and the first and most common cause of human aspergillosis [77]. The review by Abad et al., on the multifactorial virulence of this fungus, presented data that associates the fungal structure and metabolic changes with its capacity to grow and adapt to stress conditions, evading the host’s immune system and causing disease [81]. *A. fumigatus* has several inherent characteristics that make it a primary pathogenic fungus: small-sized conidia (2–3 μm in diameter) that make it easier to reach the host pulmonary alveoli, thermotolerance (able to grow at 55 °C and survive at more than 75 °C, with changing proteome depending on temperature), resistance to oxidative stress, high growth rate, nutritional versatility, and a complex proteome [81]. Furthermore, its ability to cause disease is directly linked with the host environment and its immune responses [81].

In the space-based study by Sielaff et al. [16], eighty-one fungal strains were isolated from the ISS surfaces. These were identified by Sanger sequencing of the ITS region, which revealed that the predominant species was *Rhodotorula mucilaginosa*, followed by *Penicillium chrysogenum*, both capable of causing human infections [82,83].

*Aspergillus niger* is another very well studied fungal species that was able to survive and thrive on board the ISS, exposed to microgravity, enhanced radiation, and low nutrient availability. This species, highly relevant for its biotechnological applications, has been reported as an opportunistic human pathogen [84]. Romsdahl et al. [18] isolated this species from ISS surfaces, and after extensive analysis found it to have a different secondary metabolite profile. It presented an increased production of pyranonigrin A, a molecule with antioxidant and UV-protective properties, which could explain the resistance of this strain to the ISS environment conditions [18].

Many studies [15,16,18,55,79] were performed on the survival, development and adaptation of fungal species to outer space environments; some researched fungal contamination of different surfaces in space environments such as the Mir station or the ISS [70,85], with a few even suggesting the need for technological development of new types of material with antimicrobial properties, so as to avoid contamination by fungi [85] and other microorganisms [86]. Part of these efforts are driven by the detrimental effect that fungal growth and activity has on equipment and materials at the ISS.

#### 1.3.3. Other Microorganisms

Extremophilic archaea are a key group of microbes when discussing astrobiology and planetary protection concerns as referred by Rettberg et al. [87] and de Vera et al. [88]. Understandingly, several authors have looked into their resistance as part of exposure experiments (e.g., exposing the methanogenic archaeon *Methanosarcina* sp. to simulated Mars conditions [88], the halophilic archaeon *Halorubrum chaoviator* to simulated and real space conditions [89], and the halophilic archaea *Halobacterium salinarum*, *Halococcus morrhuae*, and *Halococcus hamelinensis* to simulated solar radiation [90]). After exposure of *Haloferax mediterranei* and *Halococcus dombrowskii* to simulated microgravity, several phenotypic and genotypic changes were observed, including increased antibiotic resistance, differences in pigmentation and in their proteomes [91]. Exposure of other haloarchaeal strains from different species to simulated solar radiation, showed different species-dependent responses: some did not tolerate increased radiation (*Halococcus hamelinensis*) but accumulated compatible solutes that facilitated after-exposure survivability, some had higher survivability rates probably due to cell clustering (*Halococcus morrhuae*), and others had higher DNA-repairing rates during exposure (*Halobacterium salinarum*) [90]. Outside extreme settings, Archaea are often overlooked as they have little relevance from a microbial pathogenicity perspective. While it is true that no archaeal pathogen has been identified thus far, they are increasingly seen as important components of several microbiomes, including our own [92], so new developments on the effects of their interactions within our microbiome are highly likely.

The study of viruses in a space context remains underexplored but is expected to gain increased attention, namely as the object of study of astrovirology [93,94]. Only a few studies have looked into virus stability in the context of space exploration, tested with exposure to simulated space-like conditions, high-altitude balloons and rocket launches, or on-board experiments at the ISS. Tested viruses include e.g., bacteriophages T1 and T7, the tobacco mosaic virus, and poliovirus with minimal infectivity losses [94,95,96]. It is worth noting that viruses are the only known biological entities with a “phoenix phenotype”: infection of multiple damaged viruses can restore replication capability if they have an equivalent to a full, undamaged genome [93].

#### 1.3.4. Examples of Studies Made So Far to Understand the Space Adaptability of Microorganisms

Understanding microbial pathogenicity is still an ongoing process, but many studies (Table 2) have contributed to clarifying possible changes and potential developmental evolution that might accompany present and future explorations in space.

### 1.4. Can There Be Any Dangers of Exposure and Return to Common Environments?

Most of the studies, experiments, and analyses undertaken so far, focus on what happens, or might happen, when organisms are exposed to different and stressful conditions. However, another issue is: what happens when such organisms, adapted to conditions we consider as unusual and stressful, are transferred to so-called “regular” conditions (e.g., what happens when microbes adapted to the space environment are brought back to Earth conditions?). This question can be applied to microbes returned from extreme environments, either originating from those extreme settings or to those originated from non-extreme settings but adapted after exposure to stressing environments. Will adapting microbes cope better than the ones that are re-adapting? Could non-pathogenic microorganisms fully adapted (able to develop, grow and multiply) to space conditions become pathogenic once returned to Earth where its development would be easier? How are host-microorganism interactions affected? Such considerations become more and more relevant with the increase of space exploration but the amount of collected data on this still remains scarce.

Ryba-White et al. [109], tested soybean seedlings under spaceflight conditions and compared these with others, grown on Earth, under regular conditions. The soybean roots were inoculated with the phytopathogenic fungal species *Phytophthora sojae*. Those exposed to space flight conditions, presented a more extensive infection, noted with an increased number of fungal structures present on the roots; but also presented root length similar to non-inoculated roots, and longer with higher number of lateral roots than the inoculated roots grown on Earth. The results obtained point to a higher susceptibility to infection, where the fungus became more pathogenic to the seedlings exposed to microgravity [109]. Nevertheless, this study focused on the host and its changes after microgravity exposure, and not on the pathogen. Further analysis to understand any possible changes on the fungal strain would have provided some important insights into this topic.

Gilbert et al. [17] have also noted an increase in virulence of *Serratia marcescens*, after growth on the ISS or when grown in a simulated microgravity rotating wall vessel on Earth, when compared to ground-based controls. However, after a few generations back on Earth, this phenotypic virulence was lost [17].

Given this, microbial communities need to be fully accessed and controlled or even eradicated from certain environments such as spacecrafts and clean-rooms where space materials are assembled. Such precautions are meant not only to control potential forward and backward contamination, but also to minimize any risks of potential development of diseases and infections during space missions. Rigorous sterilization and disinfection processes can be performed by several different methods, using chemicals (e.g., ethylene oxide, hydrogen peroxide, nitrogen dioxide, chlorine dioxide, peracetic acid), radiation (ionizing or ultraviolet), high temperatures (steam or dry heat) [110]. More recently developed methods, such as the use of low-temperature plasma (ionized gas), are also being adapted and used for these purposes [111]. Furthermore, in order to contain microbial growth, the use of materials with antimicrobial properties (e.g., with inclusion of metallic nanoparticles) is also being increasingly analysed and implemented [112].

### 1.5. What Are the Effects and Impact of Space Exposure on Humans?

As discussed for microbes, humans are also exposed to a variety of hostile environmental changes during spaceflight. Those include variations in gravity (from microgravity to hyper-gravity periods, which occur during launching and landing), acute and chronic exposure to radiation, and psychological stress, as well as loss of nycthemeral cycles and perturbation of normal circadian rhythms, exposure to extreme temperatures, variable magnetic fields, and hypercapnic conditions ([5,17,113,114,115]). In more prolonged missions, long-term isolation impairs sleep, mood, and alertness, compromises muscular strength and endocrine physiology, induces changes in hormone levels and metabolism [17,116], and causes microbiome shifts [9]. At the molecular level, we see changes in oxidative stress, DNA damage, mitochondrial dysregulation, epigenetics (including gene regulation), and telomere length (reviewed in [9]).

Spaceflight stressors affect many physiological systems and lead to direct and indirect impacts in the immune system, with effects both in acquired and innate immunity [17,117]. This detrimental effect on astronauts’ immunity likely explains the increase in opportunistic infections (e.g., viral by cytomegalovirus, varicella-zoster virus, or Epstein–Barr virus [114]; bacterial, such as conjunctivitis and acute respiratory and dental infections [118]), both during their time in space and a short period of time after their return to Earth [114,117,119,120,121,122]. Furthermore, the synergistic effects of prolonged microgravity and other space flight stressors may exacerbate complex health problems in astronauts [9].

Microgravity induces adaptations at the cellular and molecular level with genomic, epigenomic, and proteomic changes, which create risks for a range of pathologies [9]. Microgravity is the stressor considered as responsible for the higher number of body changes. These include loss of bone and irreversible bone resorption, loss of muscle mass, cardiovascular deconditioning and dysfunction, impaired exercise capacity, altered neurovestibular perception, ocular changes, immune-deficiency, alterations of peripheral metabolism) [5,9,120]. Altered gravity affects biological processes that respond to normal gravity, causing abnormal physiological responses. A typical example is what is observed with bodily fluids such as blood, which shift upward toward the head and thorax, leading to decreased leg volume and compensatory cardiovascular system changes [9,123]. Microgravity also seems to be the major factor of space flight responsible for dysregulation of the immune system and increased clinical risk as analysed on the host-pathogen experiences, with flies *Drosophila melanogaster* (infection model for *Serratia marcescens*) showing an increased virulence of the space exposed *S. marcescens* [17] and with zebrafish which present a decreased antiviral immunity after exposure to simulated microgravity [120]. Most of the research has uncovered changes in the number of immune cells and on immunological memory; but, few studies looked into cytokine production in response to antigens or activation of innate immunity [124,125].

#### 1.5.1. Development of Acquired Immunity

Lymphocytes play key roles in immunity. As briefly summarized in [113], invasion by pathogens activates the innate immunity (namely monocytes/macrophages), which triggers T lymphocyte activation and differentiation via antigen presentation and activity of several cytokines. Activated effector memory T cells (e.g., CD4+ and CD8+ lymphocytes) further cascade this, namely by activating B lymphocytes and macrophages. After pathogens removal, some of these antigen-specific B and T lymphocytes are converted into immunological memory cells, thus contributing to long-lasting immunity.

**Figure 2:**
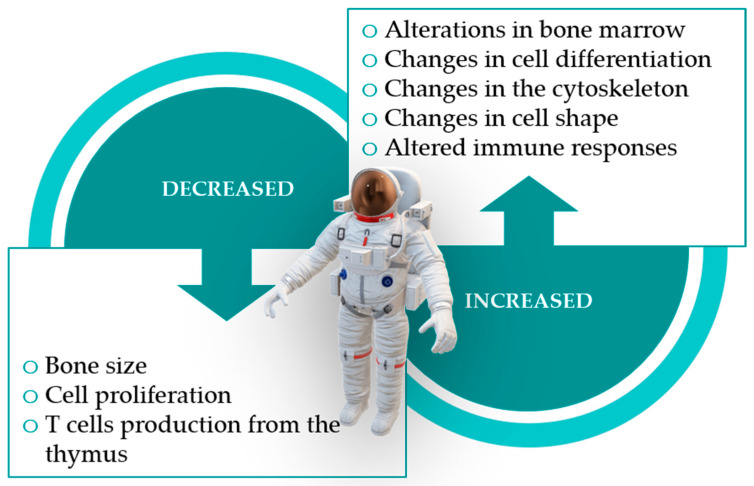
Impact of microgravity and space radiation on astronauts.

Spaceflight appears to have an impact on the development of acquired immunity as it affects lymphoid organ homeostasis and the differentiation and maturation of several cells of the immune system, particularly lymphocytes [120,125]. It is worth noting that B lymphocytes, myelocytes, erythrocytes, hematopoietic stem cells and other progenitor cells are formed and mature in the bone marrow, and that bones suffer some of the most drastic changes when under microgravity (Figure 2), with sharp decreases in their mass [5,9,113,120]. Mesenchymal stem cells, which are also vital in forming the bone marrow microenvironment, also seem to suffer changes in their differentiation induced by microgravity and space radiation [113].

The thymus, another primary lymphoid organ responsible for the production of almost all T lymphocytes, is known to undergo atrophy when exposed to various physiological and psychological stressors [113]. It is thus unsurprising that studies report on thymic atrophy during spaceflight and suggested impairment of T lymphocyte development [113,126].

#### 1.5.2. Immune Cell Responses

In addition to the effects on production and development of immune cells, spaceflight also appears to impact immune cell responses against pathogens, allergens and tumours [113]. Reported changes include: variations in regular levels of many cells, in peripheral parts of the body and their overall distribution; changes in function of e.g., granulocytes, monocytes, and natural killer cells; significant reduction in activation of T lymphocytes; and, changes in cytokine levels in plasma and in response to stimuli [11,113,114,124]. Such effects extend to the post-flight period, with tests on astronauts showing a temporary reduction in phagocytic capability and an attenuated oxidative burst and degranulation by neutrophils and monocytes [11,114].

Spaceflight stressors also activate the hypothalamic–pituitary–adrenal and sympathetic–adrenal–medullary axes and increase the levels of stress hormones such as cortisol, dehydroepiandrosterone, adrenaline, or noradrenaline. These hormones may be partly responsible for the aforementioned changes given their recognized impact in modulating immune cells [113].

Other authors suggest a potential role of microgravity-induced changes in cytoskeletal properties, with decreased expression of surface receptors and motility, which would hamper interactions and compromise immune cell activation [119,126].

#### 1.5.3. Immunity and Onset of Disease

There are currently no known reports of astronauts being afflicted by serious infectious diseases during or immediately after space flight. According to some, this could be associated with the general physical and mental robustness and fitness of astronauts and the lack of cases with very extended exposure to space flight [113]. The dawn of commercial space flight will significantly widen this limited dataset, providing a more inclusive and representative pool of humans [9,114].

Despite the aforementioned lack of serious diseases, there are several reports of reactivation of latent viral infections (most notably human herpesviruses, Varicella–Zoster virus, and Epstein–Barr virus) before, during, and after spaceflight [113]. This reactivation is associated with increased cortisol levels and decreased interferon production, suggesting a link with stress [114].

Spaceflight might also affect the onset and progression of allergies and autoimmunity, with reported increases in cases of allergy-like symptoms, rashes, and hypersensitivity [113]. These suggest dysregulation of immune and inflammatory response but the mechanisms behind this remains unclear and some authors even propose a potential link with changes in astronauts’ microbiomes [113,127]. Human space exposure, and all the arising changes, facilitate pathogenic infections. Moreover, for some potential pathogens, their ability to cause disease is directly related with the host, its immune system and its immune system response [81].

#### 1.5.4. Changes in Microbiome

Exposure to spaceflight has an apparent effect on the astronauts’ microbiomes, with changes in overall diversity and shifts in ratios between different taxa, impacting host–pathogen interactions [5,9,127]. These microbiome changes may be due to a direct impact of exposure to microgravity (and other environmental changes occurring during spaceflight) on microbes, or the indirect result of spaceflight affecting the hosts and their physiologies, including via increased stress and changes in diet [5].

Other important factors to take into consideration here, are the impacts of microbial transference between crew members and between them and surfaces of their confined environments, and their tendency towards homogenization in such a closed setting [7].

#### 1.5.5. Limitations and Countermeasures

The study of the effects of spaceflight on humans suffers from some well-known limitations. For example, it is recognized that there is wide variation in the effects of exposure to space flight among astronauts, which are most likely linked with genotypic and phenotypic differences [5]. Also, the exact impact of spaceflight on specific human organs and physiological systems is hard to assess and often needs to rely on the use of ground-based experiments and/or the use of model organisms [113]. Based on such approaches, cellular responses are now clearer, yet intracellular regulatory mechanisms remain mostly obscure [127]. Furthermore, given that spaceflight induces many stressors and environmental changes that affect parts of an interconnected system, the individual effect of each one is not always clear.

It is also worth noting that only very limited data exist on long-term periods spent in space, as only a few people have spent more than a year in space. The record for continuous confinement in space is currently held by the cosmonaut Valery Polyakov, who spent 437 consecutive days on Mir, while the astronaut Peggy Whitson has spent 665 cumulative days over several missions [128,129]. A better understanding of the effects of spaceflight on humans is essential, to elucidate the paths of disruption, identify possible countermeasures to reduce such detrimental effects, and is vital before embarking on deeper space exploration [127].

The exposure to increased, Earth-like gravity seems to be the most adequate way to alleviate most of the issues and conditions identified in the previous sections (Figure 2), and could further improve with complementary measures such as e.g., exercise, stress management and prebiotic and probiotic intake [113,121,122].

## 2. Overview and Future Developments

As advocated by many, and from a human perspective, microorganisms could serve the roles of friends and foes both in space as on Earth. There are several studies showing that when in space, microorganisms are frequently able to grow better, reaching higher concentrations, and have more active responses to stressors, developing increased tolerance to antimicrobials increased virulence and better microbial aptitude to cause infection (e.g., [55,100,130,131]).

Therefore, this review highlights the need to fully understand microbial behaviour, development and evolution in space conditions. Multidisciplinary research is a must in order to address the issue of pathogenicity in space, to avoid Earth’s non-pathogenic microorganisms from gaining pathogenicity traits once in space, to avoid increased pathogenicity of Earth’s pathogens, to avoid humans developing a frailer immune system with higher infection rates during space exploration, and to develop protective systems to guaranty the health and safety of space crews.

## Figures and Tables

**Table 1:** Outer space parameter and resultant microbial alterations and/or adaptations.

Parameter	Alterations and/or Adaptations	Reference
Solar UV radiation	Increased microbial mutation rates. DNA damage through double/single-strand breaks, base modifications or pyrimidine dimerization. Oxidative stress which induces the production of key enzymes (e.g., catalase).	[8,12]
High vacuum	DNA damage through induction of base deletion and insertion.	[8]
High-dose ionizing radiation or desiccation	DNA damage through double-strand breaks (DSBs).	[8]
Radiation capable of penetrating the International Space Station (ISS) spacecraft	Generation of reactive oxygen species (ROS) within biological systems, with oxidative stress and consequent DNA damage.	[6,18]
Microgravity	Increased growth. Smaller lag phase in bacterial growth curves. Shear forces get reduced, there is no sedimentation, diffusion processes get slower, and no convection without gravity. Hindered access to oxygen, metabolites, and nutrients, affecting mass and heat transfer.	[6,12,60]

**Table 2:** Examples of exposure and survivability experiments in outer space environment.

Organism Exposed	Exposure Experiment		Results and Conclusions Obtained	Reference
	Location	Time		
Axenic and mixed cultures of microorganisms capable of essential nitrogen cycle conversions	Low Earth Orbit (LEO, 258–571 km of altitude), on a Foton-M4 flight	44 days	▪ There was a similar or increased nitrogen conversion. ▪ Refrigerated space exposure suggested maximum microbial reactivation.	[97]
*Bacillus* sp. (two psychrotolerant strains); *Bacillus horneckiae*, and *Bacillus licheniformis* (thermophiles)	HIMAC at the NIRS, Japan, with exposure to HZE particles: He and Fe ions, presence of germinants (Glu, Ala, and Val).	0–8 h	▪ Bacterial spores survived He irradiation, but showed low viability when exposed to Fe ions. ▪ Thermophilic spores survived better than psychrotolerants. ▪ Germination kinetics varied depending on the type/dose of irradiation and the germinant used. ▪ Spores’ germination efficiency was altered, it increased after He irradiation.	[98]
*Bacillus subtilis*, *Cupriavidus metallidurans*, and *Sphingomonas desiccabilis*	Simulated Martian gravity (0.38× *g*), on the ISS	21 days, plus 2 days-flight (on a Space X Falcon-9 rocket)	▪ The cell counts and optical density measured were similar to the ones obtained for microgravity and Earth’s gravity.	[99]
*Deinococcus aerius* and *Deinococcus radiodurans*, in cell pellets	Outside the ISS	1–3 years	▪ Cell pellets with 500–1000 μm of thickness survived for 3 years. ▪ Cells aggregated into pellets with a certain thickness can survive UV-radiation and survive space environment for several years.	[8]
*Escherichia coli*	Simulated microgravity, HARV	Time needed for the growth of 1000 generations	▪ Maintained resistance to chloramphenicol, and acquired resistance to cefalotin, cefuroxime, cefuroxime axetil, cefoxitin, and tetracycline. ▪ Deletion from the genome of 14 genes, involved in motility and chemotaxis.	[100]
*Humicola fuscoatra*	Space Shuttle mission STS-77	10 days	▪ Increased production of the antimicrobial monorden.	[101]
*Klebsiella pneumonia*	Outer space of the Shenzhou VIII spacecraft	17 days	▪ Multiple genomic changes. ▪ Increased diversity after spaceflight. ▪ Acquired resistance to sulfamethoxazole.	[102]
*Lactobacillus acidophilus*	Simulated microgravity (RWV)	Up to 36 h	▪ Increased growth rate, acid tolerance, bile resistance, and in vitro cholesterol-lowering ability. ▪ Decreased lag phase, and sensitivity to cefalexin, gentamicin, and penicillin. ▪ Higher antibacterial activity against *Salmonella typhimurium* and *Staphylococcus aureus*.	[60]
*Lactobacillus reuteri*	Simulated microgravity (RWV)	18 h	▪ Increased production of the antimicrobial compound reuterin. ▪ Survival rates of cells exposed to gastrointestinal stress higher than the control under terrestrial gravity. ▪ Increased expression of stress-related genes.	[37]
*Lactobacillus reuteri*	Simulated microgravity (RPM)	18 h	▪ Increased production of the antimicrobial compound reuterin. ▪ Survival rates of cells exposed to gastrointestinal stress similar to the control under terrestrial gravity. ▪ decreased expression of stress-related genes, increased expression of the genes *rex*, *map* and *msa*.	[37]
*Ralstonia pickettii* and *Sphingobacterium thalpophilium* (isolated from water systems of the Mir space station)	Simulated microgravity (STLV on a RCCS)	Up to 14 days	▪ *S. thalpophilium* did not show any alterations. ▪ *R. pickettii* presented increased growth rate in high-substrate medium.	[103]
*Rhodospirillum rubrum*	Anaerobiose at ISS	8 days, plus 2 days flight (in the Soyus carrier rocket)	▪ Changes in gene expression when exposed to environmental change and grown in minimal medium.	[35]
*Rhodospirillum rubrum*	Simulated microgravity (RPM) and space-ionizing radiation, on Earth	10 days	▪ Increased alterations in a simulated environment with rich medium, when compared to ISS conditions.	[35]
*Rhodospirillum rubrum*	Simulated partial microgravity (RWV) conditions, on Earth	10 days	▪ Significant alterations at the transcriptomic, proteomic and metabolic levels (higher pigmentation, increased production of some metabolites, upregulated genes.	[104]
*Serratia marcescens*	Spaceflight conditions at ISS	n.r.	▪ Higher bacterial growth rate observed. ▪ Increased virulence in infected *Drosophila melanogaster* hosts, which was lost after re-growth under Earth conditions.	[17]
*Serratia marcescens*	Simulated microgravity (RWV)	n.r.	▪ Increased virulence in infected *Drosophila melanogaster* hosts, which is lost after re-growth under Earth conditions.	[17]
Spores of *Aspergillus sydowii*, *Aspergillus versicolor*, *Penicillium aurantiogriseum*, and *Penicillium expansum*	Outer surface of ISS	Over 22 months	▪ All fungal spores survived UV irradiation, and *P. aurantiogriseum* spores presented the lowest survival rate. ▪ Antimicrobial resistance for *A. versicolor* and *Penicillium* spp. increased with higher values of UV irradiation. ▪ Antimicrobial resistance for *A. sydowii* increased at the lower values of UV irradiation.	[55]
Spores of *Bacillus licheniformis*, *Bacillus pumilus*, and *Bacillus subtilis*	Outer surface of ISS	Over 22 months	▪ No viability at 100% transmission of UV irradiation, but some reduced viability at 1% transmission for *B. pumilus* and *B. subtilis*, which increased at lower transmission values. ▪ *B. subtilis* was the most susceptible strain to UV. ▪ UV transmission >1% led to the loss of RNase activity and decrease of RNase activity. ▪ Decrease of antimicrobial resistance for all strains tested.	[55]
Spores of *Bacillus subtilis*	Outside MIR space station.	3 months	▪ Survival of cells when protected from radiation.	[20]
*Staphylococcus warneri*	Space environment aboard Tiangong-2 space laboratory.	64 days, plus 15 days-flight (on the Shenzhou-10 spacecraft)	▪ Increased biofilm formation ability. ▪ Upregulation of genes expressing phosphotransferase. ▪ Enhanced resistance and adaptability to the external environment.	[105]
*Streptomyces coelicolor*	Shenzhou-8 spacecraft, and simulated microgravity (2D-clinostat) on Earth.	16.5 days	▪ Shorter life cycle and increased sporulation. ▪ Increased biomass in liquid cultures. ▪ Altered secondary metabolites profile, with apparent increased production of bioactive substances.	[106]
*Streptomyces plicatus*	Space Shuttle mission STS-80	7 and 12 days	▪ Reduced number of colony forming units. ▪ Increased production of the antibiotic actinomycin D.	[107]
*Streptomyces plicatus*	ISS	8, 12, and 72 days	▪ Higher number of viable cells. ▪ Increased production of the antibiotic actinomycin D at 8 and 12 days.	[108]

Ala = L-alanine; Fe = Iron; Glu = D-glucose; HARV = high-aspect-ratio vessels; He = Helium; HIMAC = Heavy ion medical accelerator; HZE = High (H) Charge (Z) Energy (E); ISS = International Space Station; n.r. = not reported; NIRS = National Institute for Radiological Sciences; RPM = random-positioning machine; RWV = rotating wall vessel; SLTV = slow turning lateral vessels; RCCS = rotary cell culture system; Val = L-valine.

## Data Availability

Not applicable.
